# Application of the UK Foresight Obesity Model in Ireland: The Health and Economic Consequences of Projected Obesity Trends in Ireland

**DOI:** 10.1371/journal.pone.0079827

**Published:** 2013-11-13

**Authors:** Laura Keaver, Laura Webber, Anne Dee, Frances Shiely, Tim Marsh, Kevin Balanda, Ivan Perry

**Affiliations:** 1 Department of Epidemiology and Public Health, University College Cork, Cork, Ireland; 2 UK Health Forum, London, United Kingdom; 3 Health Service Executive Department of Public Health, Limerick, Ireland; 4 Institute of Public Health in Ireland, Dublin, Ireland; Virginia Tech, United States of America

## Abstract

**Background:**

Given the scale of the current obesity epidemic and associated health consequences there has been increasing concern about the economic burden placed on society in terms of direct healthcare costs and indirect societal costs. In the Republic of Ireland these costs were estimated at €1.13 billion for 2009. The total direct healthcare costs for six major obesity related conditions (coronary heart disease & stroke, cancer, hypertension, type 2 diabetes and knee osteoarthritis) in the same year were estimated at €2.55 billion. The aim of this research is to project disease burden and direct healthcare costs for these conditions in Ireland to 2030 using the established model developed by the Health Forum (UK) for the Foresight: Tackling Obesities project.

**Methodology:**

Routine data sources were used to derive incidence, prevalence, mortality and survival for six conditions as inputs for the model. The model utilises a two stage modelling process to predict future BMI rates, disease prevalence and costs. Stage 1 employs a non-linear multivariate regression model to project BMI trends; stage 2 employs a microsimulation approach to produce longitudinal projections and test the impact of interventions upon future incidence of obesity-related disease.

**Results:**

Overweight and obesity are projected to reach levels of 89% and 85% in males and females respectively by 2030. This will result in an increase in the obesity related prevalence of CHD & stroke by 97%, cancers by 61% and type 2 diabetes by 21%. The direct healthcare costs associated with these increases will amount to €5.4 billion by 2030. A 5% reduction in population BMI levels by 2030 is projected to result in €495 million less being spent in obesity-related direct healthcare costs over twenty years.

**Discussion:**

These findings have significant implications for policy, highlighting the need for effective strategies to prevent this avoidable health and economic burden.

## Introduction

Overweight and obesity have become one of the most prominent public health issues in society today, affecting countries at every stage of development. The latest estimates indicate approximately 1.46 billion people globally are overweight and 502 million of these obese [1]. The problem is rapidly penetrating into childhood with 42 million of those under the age of five estimated to be overweight globally [2]. 

The most recent representative data for Ireland shows that 37% of the Irish adult population are overweight and a further 24% are obese [3]. This mirrors results from the Irish National Lifestyle Surveys (SLÁN) in 2007 [4]. In addition levels of childhood obesity in Ireland have been shown to be high when compared with our Northern European counterparts [5]. Preliminary results from a national pre-school nutrition survey indicate that 23% of those aged 2-4 years old are overweight or obese [6]. As a significant correlation exists between childhood and adolescent BMI and adult overweight and obesity, these current childhood obesity rates have the potential to contribute hugely to the health and economic burden of obesity in Ireland in the future [7,8,9]. 

A greater recognition of the burden of disease associated with obesity has come about in recent years [10]. This health burden is largely driven by increased incidence of type 2 diabetes, cardiovascular diseases and some forms of cancer [11]. Both the direct and indirect healthcare costs associated with these present a significant financial burden to both public health expenditures and to the state [12]. A particularly detailed recent study in Ireland has estimated total costs attributable to obesity at 1.13 billion euro [13]. Of this €399 million were direct and €729 million were indirect costs. Direct costs refer to in-patient and day case costs, out-patient costs, GP costs and drug costs. Indirect costs are more difficult to collate and in this instance referred to productivity losses associated with work absenteeism and premature mortality. Social, psychological and emotional costs were omitted from indirect cost calculations due to a lack of national data on the economic cost of these variables.

 Given the scale of the obesity epidemic there is interest nationally in determining the extent and future consequences of this issue. The UK foresight obesity model [14] has previously been applied in over 70 countries worldwide as well as each US state. Applying this model to the Irish population will provide policymakers with an idea of the potential scale of the obesity epidemic should it continue unabated, its consequences for the Irish health system, as well as the potential effect that particular policies may have if implemented. This will help guide resource allocation, population health planning and the development of effective obesity prevention and health promotion programmes to curb obesity levels within the country.

## Methods

### Data sources

The data sources on which the model’s input parameters are based can be seen in Table 1. BMI data for Ireland was collected by conducting a search of the literature using Pubmed and Google scholar and through discussion with academic departments and other relevant national stakeholders. Databases were included if BMI data was nationally representative, presented by age and sex and categorised by WHO cut-offs for normal weight (<25kg/m^2^), pre-obesity (25-29.99 kg/m^2^), and obesity (≥30kg/m^2^). Measured data was preferred however, due to lack of availability, some sources of self-reported data were included. Outliers were removed where data fell outside of 95% confidence limits.

**Table 1:** Sources of data inputs for Ireland.

**Category**	**Source of data**
**Population characteristics**	
	National Adult Nutrition Survey (2011) [21]
	SLAN (2007 [22], 2002 [23], 1998 [24])
BMI distribution	North South Ireland Food Consumption Survey (2001) [25]
	The Irish Longitudinal study on Aging (2011) [26]
Population size	Central Statistics Office (2010) [27]
**Incidence/Prevalence**	
CHD	Coronary Heart Attack Ireland Register (2006) [28]
Stroke	North Dublin Population Stroke Study (2007) [29]
Cancer	National Cancer Registry (2012) [30]
Hypertension	Health Survey for England (2010) [31]
Type II diabetes	Balanda et al., (2012) [32]
Knee Osteoarthritis	Institute of Public Health (2012) [33]
**Survival**	
CHD	European Cardiovascular Disease Statistics (2012) [34]
Stroke	Wolfe et al., (2011) [35]
Cancers	National Cancer Registry (2010) [30]
**Relative risks of obesity on disease risks**	International Association for the Study of Obesity (2010) [36]
**Disease-specific mortality**	
Coronary heart disease	Central Statistics Office (2010) [27]
Stroke	Central Statistics Office (2010) [27]
Cancer	National Cancer Registry (2009) [30]
**Healthcare costs**	
Direct	Health Atlas Ireland, HSE (2009) [37]

A review of epidemiological studies was undertaken to find incidence, prevalence, and annual healthcare costs for each obesity-related disease: type 2 diabetes, coronary heart disease, stroke, knee osteoarthritis, hypertension and obesity-related cancers (breast, colorectal, oesophageal, endometrial, liver, kidney and pancreas) by age and gender. The relative co-morbidity risks related to being overweight or obese were obtained for each of these diseases from the International Association for the Study of Obesity [15]. Mortality and survival data were also collected for fatal diseases: CHD, stroke, cancers. 

Incidence or prevalence data were not available for hypertension so UK prevalence data were used as a proxy. Survival data were not available for CHD or stroke so again the UK was used as a proxy to estimate survival in Ireland. These figures were adjusted for Irish population data [16]. 

Hypertension, Type 2 diabetes and knee osteoarthritis prevalence were converted to incidence using WHO Dismod equations [17]. 

### Statistical analysis

A two-part modelling process was used to project the future obesity-related disease burden in Ireland. The first module fits multivariate, categorical regression models, to past and current cross-sectional BMI data, to project obesity rates to 2030 by sex. Age was included as a covariate and the predicted proportions of population in each BMI category was constrained to always total 100%. 

Using the BMI levels predicted in module one, and assuming that an individual’s BMI status in the same-age cohort is consistent over time, module two simulates the BMI trajectory of the Irish population as it ages. A BMI value is probabilistically and stochastically assigned as a function of age, sex and calendar year. This results in a longitudinal growth model of the Irish population. The data collected on incidence and prevalence of disease, mortality and survival enabled the consequences of these BMI trends to be determined e.g. the modelling of each individual’s chance of contracting, surviving or dying from these set of conditions. The subsequent healthcare costs associated with these trends could then be determined. The software used in this program was written in C++. Five million Monte Carlo simulation runs were performed. 

The micro-simulation also modelled the effects of constraints on future BMI growth. By anticipating the outcome of interventions on BMI prevalence and its effects by age, year and sex, it was possible to explore how levels of obesity-related chronic disease, mortality, and healthcare costs would alter following such interventions. Three different trend scenarios were estimated: scenario 0: obesity trends continue unabated; scenario 1: population BMI decreases by 1% and scenario 2: population BMI decreases by 5%. Greater detail of this methodology is described in [11,14] and Appendix S1. 

### Ethical approval

Ethical approval was granted by the Clinical Research Ethics Committee of University College Cork. The data utilised came from established databases where data encryption was already in place and subjects could not be identified. All data used remained confidential. Consent was given by the participants of the various studies at the point of data collection for this data to be stored and used for research.

## Results

### BMI distribution

**Figure 1:**
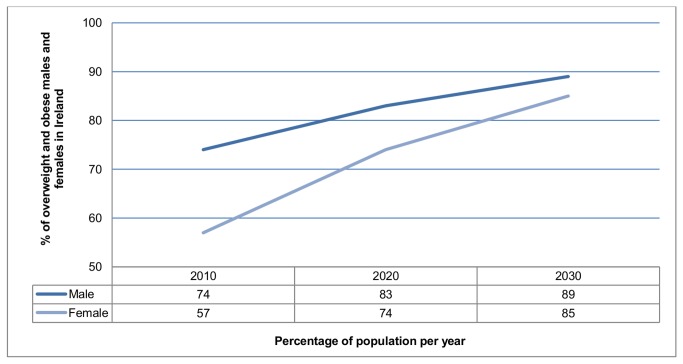
Projected prevalence of overweight and obesity in the Irish population. This figure shows the percentage prevalence of overweight and obesity for males and females in Ireland in 2010 and projected prevalence for overweight and obesity for males and females for 2020 and 2030.

The projected prevalence of overweight and obesity (≥25kg/m^2^) in males and females aged 20+ in Ireland is illustrated in Figure 1. In both cases overweight and obesity are projected to increase by 2030. At this point 89% of males and 85% of females are predicted to be overweight or obese, accounting for 3.5 million of the population. There will be an additional 717 950 overweight or obese adults in 2030 when compared to 2010. Of these additional cases 133 500 (19%) will be over 65 years of age. While males will have higher rates of overweight or obesity (≥25kg/m^2^) than females they will have lower overall rates of obesity (≥30kg/m^2^) (48% in males compared to 57% in females). Table S1 in Appendix S2 presents the proportion of the population in each BMI group by sex and age group projected to 2030. Figures S1 and S2 in Appendix S3 illustrate a sample of the projections with confidence limits. Childhood obesity was not projected due to a lack of representative data. 

All inputs into the model represent total population figures while all outputs represent the obesity related figures.

### Prevalence of disease

**Figure 2:**
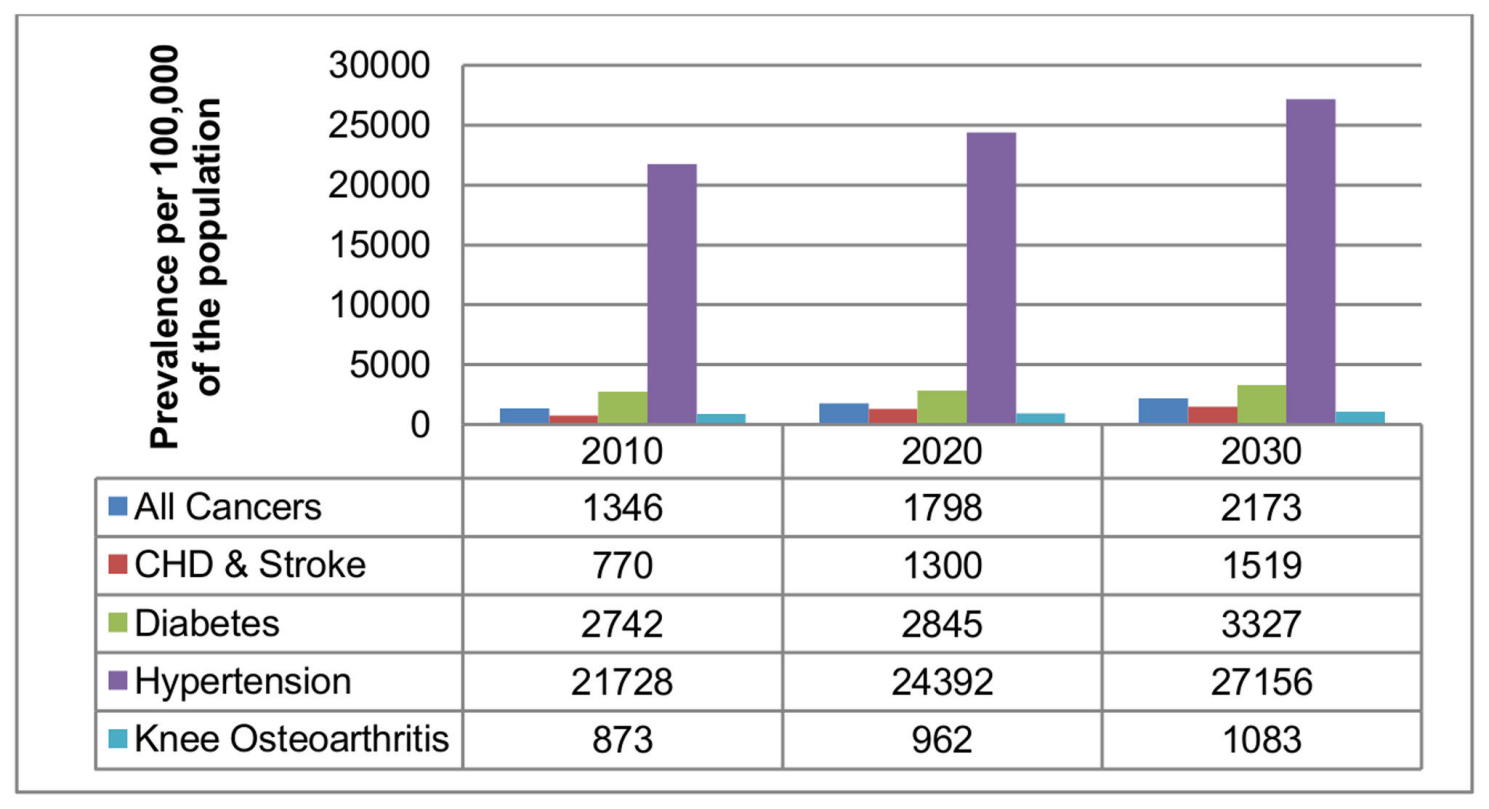
Projected prevalence of disease per 100,000 population from 2010-2030. This figure shows the projected prevalence of disease per 100,000 population for 2010, 2020 and 2030 for six obesity related conditions. These six conditions are: cancer, CHD & stroke, type 2 diabetes, hypertension and knee osteoarthritis.

The obesity related prevalence of disease per 100 000 population from 2010-2030 is presented in Figure 2 and illustrates how the burden of disease is projected to increase overtime. Based on total population figures [16], there will be approximately 67 899 cases of CHD & Stroke, 97 133 cases of cancer and 148 717 cases of type 2 diabetes by 2030. This represents a 97%, 61% and 21% increase in obesity related prevalence respectively. In addition the prevalences of non-fatal conditions such as hypertension and knee osteoarthritis are also projected to increase (affecting 27% and 1% of the total population respectively). 

### Projected outcomes from potential interventions

**Figure 3:**
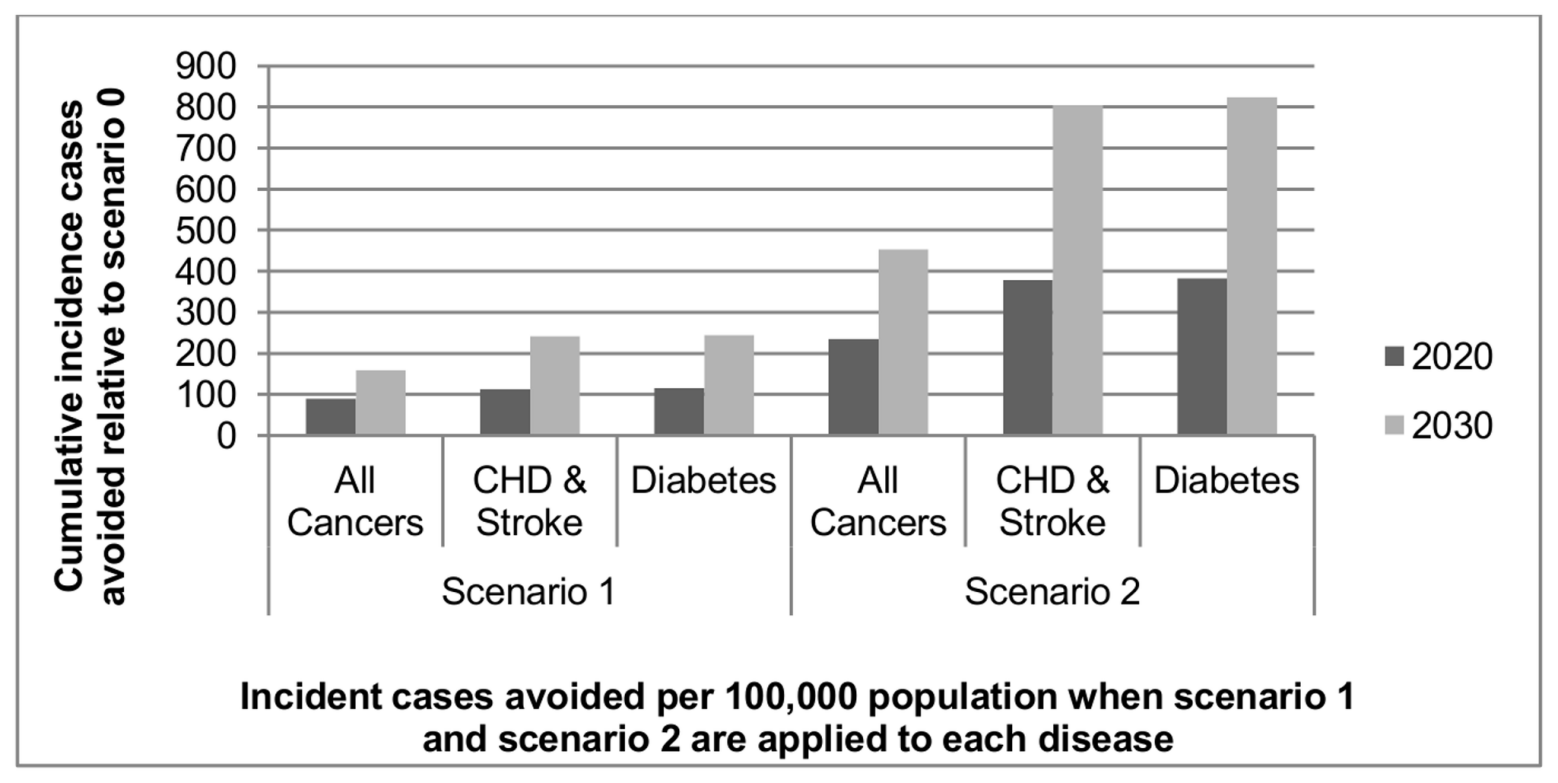
Cumulative incident cases avoided per 100,000 population relative to scenario 0 (when scenario 1 and scenario 2 are applied). This figure shows cumulative incident cases avoided per 100,000 population relative to scenario 0 when a 1% decrease in BMI is applied to the Irish population (scenario 1) and a 5% decrease in BMI is applied to the Irish population (scenario 2).

Figure 3 presents projected incident cases (obesity related) avoided per 100 000 of the population from 2010 - 2030 if, when compared to no intervention (scenario 0), a 1% (scenario 1) and 5% (scenario 2) decrease in population BMI is achieved. The cumulative incident cases avoided range from 20/100 000 in the case of knee osteoarthritis to 824/100 000 in hypertension. 

A 1% reduction in BMI in the population has a relatively small impact on disease incidence. However, it is evident that an intervention resulting in a 5% reduction in BMI levels in the Irish population would have a significant impact on the incidence of disease, particularly CHD & Stroke and Type 2 diabetes. Using total population figures this intervention is projected to decrease cumulative incidence by 35 984 and 36 788 cases respectively by 2030. The number of incident cases avoided approximately doubles between 2020 and 2030. This intervention is also estimated to significantly reduce cumulative incidence of hypertension (by approximately 2139/100 000 or 95 613 total cases within the population). A smaller though still noticeable impact is also observed on cancer incidence where 20 249 fewer incident cases could be expected. Tables S2 -S5 in Appendix S4 contains results for prevalence, prevalent cases avoided through intervention, cumulative incidence and incident cases avoided through intervention. 

### Economics

This expansion in obesity related diseases has a significant impact on healthcare costs. If obesity trends continue unabated associated direct healthcare costs are projected to reach €5.4 billion by 2030. The main contributors to these increased costs are CHD & Stroke which are projected to account for 92% of obesity-related direct costs in 2030. The increase in total direct healthcare costs from 2010 to 2030 corresponds to approximately 30.3% of total healthcare spending in Ireland in 2011. Obesity-related direct healthcare costs are presented in table 2. Tables S6-S8 in Appendix S5 present the healthcare costs by year from 2010-2030. 

**Table 2:** Direct healthcare costs by year and disease.

	**Scenario 0**			**Scenario 1**			**Scenario 2**		
**Year**	**2010**	**2020**	**2030**	**2010**	**2020**	**2030**	**2010**	**2020**	**2030**
**Total costs (€ millions)**	2547	4370	5400	2547	4270	5250	2547	3980	4900
**CHD & Stroke**	2265	4010	4970	2265	3920	4840	2265	3640	4510
**Cancer**	101	152	179	101	153	176	101	149	172

Scenario 0 = if BMI trends continue unabated; Scenario 1 = a 1% reduction in BMI levels relative to scenario 0 and Scenario 2 = a 5% reduction in BMI levels relative to scenario 0.

It can be seen from table 2 that scenario 1, where a 1% decrease in BMI is achieved across the population, is estimated to lead to direct healthcare costs that are €104 million less by 2020 and €143 million less by 2030 than scenario 0. A 5% reduction in BMI population levels (scenario 2) has a more significant impact with €394 million and €495 million less projected to be spent on obesity and its related co-morbidities by 2020 and 2030 respectively. 

## Discussion

This is the first application of the UK foresight obesity model to Irish data. It is also the first to report the projected direct health care costs of six obesity related conditions, CHD & stroke, diabetes, hypertension, cancer and knee osteoarthritis. If obesity trends continue unabated, overweight and obesity is projected to account for 89% of males and 85% of females in 2030, leading to 288 315 cumulative incident cases of cancer, 542 390 of CHD & stroke, 159 490 of type 2 diabetes, 961 005 of hypertension and 46 533 of knee osteoarthritis. This is projected to result in obesity-related direct healthcare costs of €5.4 billion by 2030.

A recent comprehensive study [13] of the cost of obesity in Ireland in 2009 included some estimates of indirect costs to the economy. It has been applied here to provide a crude estimate of the indirect costs of obesity which cannot be determined using the UK foresight obesity model. This was achieved by deriving the ratio of direct to indirect costs from the recent Irish study [13] and applying it to the direct healthcare costs obtained from the foresight model. We estimate that indirect costs will reach €9.88 billion in 2030. Both current and projected indirect costs are likely to be significant underestimates due to difficulty in obtaining accurate data and the fact that the social, psychological and emotional effects of obesity which contribute to indirect costs are hard to quantify. 

This Irish research replicates work conducted in other countries using the UK foresight obesity model. The results of this study are consistent with that found in the Russian Federation, Poland, the UK, USA and Latin America [18,19,20,11], where the level of overweight and obesity is projected to increase, leading to an increased burden of disease and associated healthcare costs. 

While the US has always been significantly ahead of Europe with regards obesity prevalence, modelled trends project a prevalence of 50-51% in males and 45-52% in females to 2030 in the US [11]. These figures are very similar to obesity rates projected for Ireland (48% in males and 57% in females), suggesting we may catch up with the US by 2030. It is important to note that there have been plateau’s observed in obesity levels in a selection of countries worldwide, were these to occur in Ireland they would alter these projected trends beneficially. 

### Strengths and Weaknesses

There are many unique aspects to this model; it is modular in nature and capable of estimating the future effects of current interventions. In addition it has the ability to process and assimilate a wealth of data.

Like any research it also has its limitations. Recent increases in childhood obesity have not been incorporated into the projected adult obesity rates due to a lack of representative data. Therefore the true prevalence of obesity in 2030 and beyond is likely to be higher than that projected by the UK foresight model. In addition, the direct healthcare costs of childhood obesity have not been included in the burden calculations. 

The projections can only be as accurate as the data input into the model and in many cases good quality healthcare data is difficult to locate. Drug costs for cancer were not included as recorded national data did not specify the cancer site or type. UK proxies for hypertension prevalence and CHD & Stroke survival were used in this paper due to unavailability of Irish data in the desired format, highlighting the need for increased health surveillance in Ireland. There are too few data points available for Ireland but the estimates are still within 95% CI and reliable up to 2020. 

These projections are based on BMI trends in Ireland over the last decade and recent past trends do not always predict longer term future trends. While obesity has been increasing at a steady pace over recent decades [1], future trends can be shaped by new environments and technologies that cannot be foreseen. 

## Conclusion

In this research we modelled three scenarios projecting the consequences of no action and the impact of two separate interventions to reduce BMI within the population. When looking at healthcare research it is not the total cost of illness that is important but rather determining if that cost is avoidable. In the case of obesity the related disease burden and subsequent healthcare costs are avoidable. 

It is clear that a 5% reduction in overweight and obesity (≥25kg/m^2^) levels will have a significant cost saving effect (€495 million less being spent in obesity related direct healthcare costs over the next twenty years). Governments, health policy makers, public health personnel and the food and drinks industry will now need agreement on how best to achieve this attainable target. Ignoring this inevitable demise is not an option if we have the interests of future generations at heart. 

In conclusion, this research provides evidence using a recognised forecasting model of avoidable direct health care costs for six major obesity related illnesses thereby providing all those involved in healthcare provision with a platform from which to work, as well as evidence of the likely consequence of the ‘do nothing new’ scenario. 

## Supporting Information

Appendix S1 **Technical information.**
(DOCX)

Appendix S2 **BMI proportions.** Table S1, Proportion of people in each BMI group by age and sex projected to 2030.
(DOCX)

Appendix S3 **Sample of the projections with confidence limits.** Figure S1, BMI projections for females aged 60-69. Figure S2, BMI projections for males aged 60-69.
(DOCX)

Appendix S4 **Prevalence and Incidence**.
Table S2, Prevalent cases in year [per 100000] for Ireland. Table S3, Prevalent cases avoided in year [per 100000] for Ireland. Table S4, Cumulative incidence cases from year 2010 [per 100000 of population in 2010] for Ireland. Table S5, Cumulative incidence cases avoided from year 2010 [per 100000 of population in 2010] for Ireland.
(DOCX)

Appendix S5 **Healthcare costs by year, 2010-2030.** Table S6, Scenario 0 – Obesity trends continue unabated (millions of euro). Table S7, Scenario 1- 1% decrease in population BMI. Table S8, Scenario 2- 5% decrease in population BMI.
(DOCX)
