# Correction: Application of the UK Foresight Obesity Model in Ireland: The Health and Economic Consequences of Projected Obesity Trends in Ireland

**DOI:** 10.1371/annotation/3dd20b6b-2a94-4542-a058-2cc7effe881a

**Published:** 2013-12-19

**Authors:** Laura Keaver, Laura Webber, Anne Dee, Frances Shiely, Tim Marsh, Kevin Balanda, Ivan Perry

The name of the last author is incorrect. The correct name is: Ivan J. Perry. The correct abbreviation in the Author Contributions Statement is: IJP. 

