# Supplementary material for: Application of the UK Foresight Obesity Model in Ireland: The Health and Economic Consequences of Projected Obesity Trends in Ireland
Source: PLoS One. 2013 Nov 13;8(11):e79827. doi: 10.1371/journal.pone.0079827 (PMC3827424; doi:10.1371/journal.pone.0079827)
Supplement: Appendix S2 — BMI proportions. Table S1, Proportion of people in each BMI group by age and sex projected to 2030. (DOCX) [file pone.0079827.s002.docx]

**Supplementary Information**

**Appendix 2:** BMI proportions

**Table S1:** Proportion of people in each BMI group by age and sex projected to 2030

|  | **2010** |  |  | **2020** |  |  | **2030** |  |  |
| --- | --- | --- | --- | --- | --- | --- | --- | --- | --- |
|  | <25 | 25-30 | >30 | <25 | 25-30 | >30 | <25 | 25-30 | >30 |
| **20-24, m** | 0.39 | 0.41 | 0.2 | 0.19 | 0.39 | 0.42 | 0.07 | 0.27 | 0.66 |
| **20-24, f** | 0.53 | 0.28 | 0.19 | 0.28 | 0.31 | 0.41 | 0.1 | 0.25 | 0.64 |
| **25-29, m** | 0.39 | 0.41 | 0.2 | 0.19 | 0.39 | 0.42 | 0.07 | 0.27 | 0.66 |
| **25-29, f** | 0.53 | 0.28 | 0.19 | 0.28 | 0.31 | 0.41 | 0.1 | 0.25 | 0.64 |
| **30-34, m** | 0.24 | 0.49 | 0.27 | 0.15 | 0.45 | 0.4 | 0.08 | 0.38 | 0.54 |
| **30-34, f** | 0.47 | 0.34 | 0.2 | 0.27 | 0.41 | 0.32 | 0.14 | 0.43 | 0.44 |
| **35-39, m** | 0.24 | 0.49 | 0.27 | 0.15 | 0.45 | 0.4 | 0.08 | 0.38 | 0.54 |
| **35-39, f** | 0.47 | 0.34 | 0.2 | 0.27 | 0.41 | 0.32 | 0.14 | 0.43 | 0.44 |
| **40-44, m** | 0.22 | 0.54 | 0.25 | 0.16 | 0.57 | 0.27 | 0.12 | 0.6 | 0.29 |
| **40-44, f** | 0.5 | 0.31 | 0.19 | 0.44 | 0.28 | 0.28 | 0.36 | 0.24 | 0.4 |
| **45-49, m** | 0.22 | 0.54 | 0.25 | 0.16 | 0.57 | 0.27 | 0.12 | 0.6 | 0.29 |
| **45-49, f** | 0.5 | 0.31 | 0.19 | 0.44 | 0.28 | 0.28 | 0.36 | 0.24 | 0.4 |
| **50-54, m** | 0.26 | 0.5 | 0.24 | 0.26 | 0.49 | 0.25 | 0.26 | 0.47 | 0.27 |
| **50-54, f** | 0.34 | 0.41 | 0.25 | 0.23 | 0.45 | 0.32 | 0.14 | 0.46 | 0.4 |
| **55-59, m** | 0.26 | 0.5 | 0.24 | 0.26 | 0.49 | 0.25 | 0.26 | 0.47 | 0.27 |
| **55-59, f** | 0.34 | 0.41 | 0.25 | 0.23 | 0.45 | 0.32 | 0.14 | 0.46 | 0.4 |
| **60-64, m** | 0.19 | 0.5 | 0.31 | 0.13 | 0.49 | 0.38 | 0.08 | 0.47 | 0.45 |
| **60-64, f** | 0.31 | 0.37 | 0.32 | 0.14 | 0.25 | 0.61 | 0.05 | 0.12 | 0.83 |
| **65-69, m** | 0.19 | 0.5 | 0.31 | 0.13 | 0.49 | 0.38 | 0.08 | 0.47 | 0.45 |
| **65-69, f** | 0.31 | 0.37 | 0.32 | 0.14 | 0.25 | 0.61 | 0.05 | 0.12 | 0.83 |
| **70-74, m** | 0.22 | 0.47 | 0.3 | 0.12 | 0.41 | 0.47 | 0.06 | 0.31 | 0.63 |
| **70-74, f** | 0.35 | 0.36 | 0.29 | 0.14 | 0.22 | 0.65 | 0.03 | 0.08 | 0.89 |
| **75+, m** | 0.21 | 0.46 | 0.33 | 0.08 | 0.28 | 0.65 | 0.02 | 0.11 | 0.87 |
| **75+, f** | 0.35 | 0.36 | 0.29 | 0.14 | 0.22 | 0.65 | 0.03 | 0.08 | 0.89 |
