# Supplementary material for: Application of the UK Foresight Obesity Model in Ireland: The Health and Economic Consequences of Projected Obesity Trends in Ireland
Source: PLoS One. 2013 Nov 13;8(11):e79827. doi: 10.1371/journal.pone.0079827 (PMC3827424; doi:10.1371/journal.pone.0079827)
Supplement: Appendix S3 — Sample of the projections with confidence limits. Figure S1, BMI projections for females aged 60-69. Figure S2, BMI projections for males aged 60-69. (DOCX) [file pone.0079827.s003.docx]

**Supplementary Information**

**Appendix 3:** Sample of the projections with confidence limits

**Figure S1:** BMI projections for females aged 60-69

**
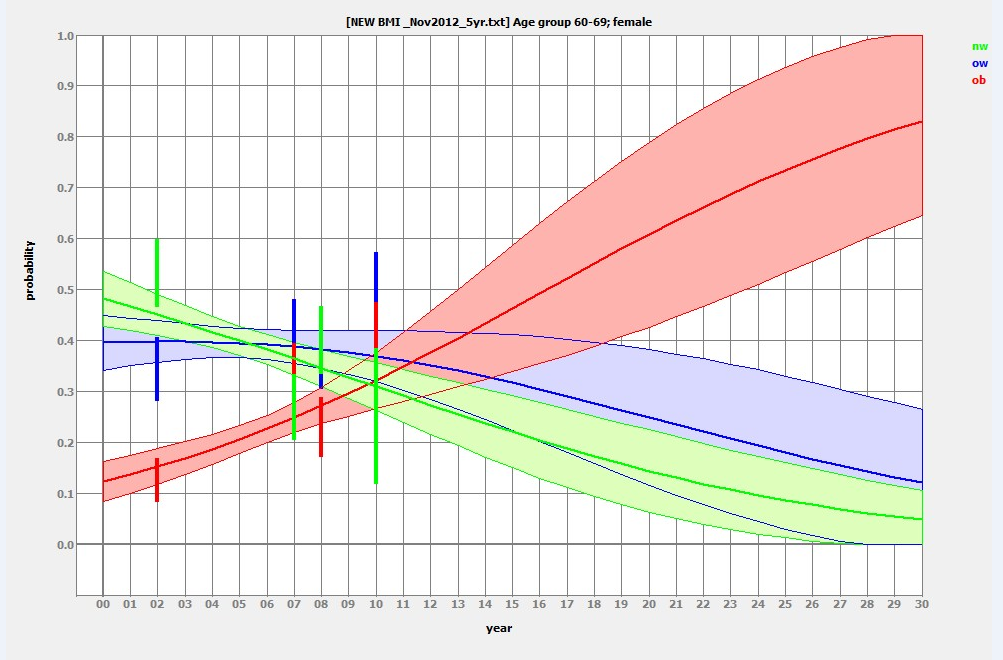
**

**Figure S2:** BMI projections for males aged 60-69

**
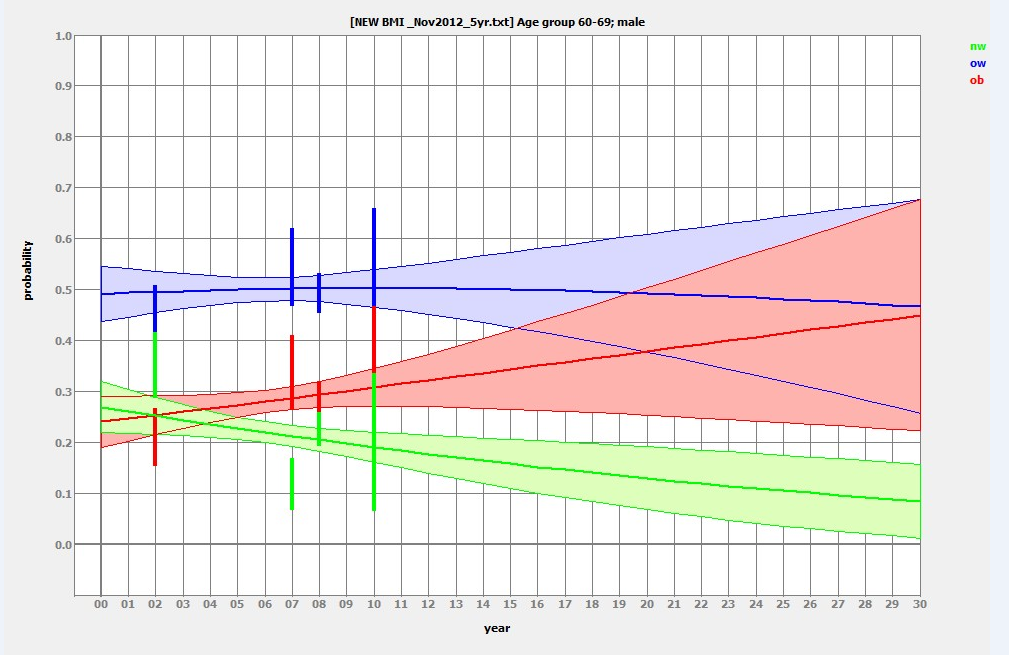
**

The above images illustrate the predicted BMI trends in males and females (aged 60-69) to 2030. These trends were derived from the regression analysis described earlier (module 1) using nationally representative data for the years 2002, 2007, 2008, 2010. The green line illustrates those who are normal weight, the blue line those who are overweight and the red line those who are obese. 95% confidence intervals are shown.

Although the 95% confidence intervals are large the estimates to 2020 are deemed reliable. Many nationally representative studies have explored BMI in Ireland but not all studies have looked at separate age groups. Figure 4 and figure 5 show the BMI projections for males and females age 60-69. Splitting these groups further into ethnic groups for example, would give too small a sample for accurate estimates.
