# Supplementary material for: Application of the UK Foresight Obesity Model in Ireland: The Health and Economic Consequences of Projected Obesity Trends in Ireland
Source: PLoS One. 2013 Nov 13;8(11):e79827. doi: 10.1371/journal.pone.0079827 (PMC3827424; doi:10.1371/journal.pone.0079827)
Supplement: Appendix S4 — Prevalence and Incidence. Table S2, Prevalent cases in year [per 100000] for Ireland. Table S3, Prevalent cases avoided in year [per 100000] for Ireland. Table S4, Cumulative incidence cases from year 2010 [per 100000 of population in 2010] for Ireland. Table S5, Cumulative incidence cases avoided from year 2010 [per 100000 of population in 2010] for Ireland. (DOCX) [file pone.0079827.s004.docx]

**Supplementary Information**

**Appendix 4: Prevalence and Incidence**

**Prevalence**

**Table S2:** Prevalent cases in year [per 100000] for Ireland

| **Prevalence cases in year [per 100000] for Ireland** | | | | | |
| --- | --- | --- | --- | --- | --- |
| Year | Cancers | CHD & Stroke | Arthritis | Diabetes | Hypertension |
| **Scenario 0** |  |  |  |  |  |
| 2010 | 1346 [+10] | 770 [+8] | 873 [+8] | 2742 [+15] | 21728 [+42] |
| 2020 | 1798 [+12] | 1300 [+10] | 962 [+9] | 2845 [+15] | 24392 [+43] |
| 2030 | 2173 [+13] | 1519 [+11] | 1083 [+9] | 3327 [+16] | 27156 [+46] |
| **Scenario 1** |  |  |  |  |  |
| 2010 | 1356 [+10] | 765 [+8] | 874 [+8] | 2738 [+15] | 21742 [+42] |
| 2020 | 1767 [+12] | 1280 [+10] | 958 [+9] | 2752 [+15] | 24048 [+43] |
| 2030 | 2142 [+13] | 1495 [+11] | 1076 [+9] | 3150 [+16] | 26566 [+45] |
| **Scenario 2** |  |  |  |  |  |
| 2010 | 1350 [+10] | 763 [+8] | 873 [+8] | 2752 [+15] | 21740 [+42] |
| 2020 | 1705 [+11] | 1220 [+10] | 932 [+8] | 2547 [+14] | 23511 [+42] |
| 2030 | 2079 [+13] | 1428 [+10] | 1035 [+9] | 2744 [+14] | 25706 [+44] |

**Table S3:** Prevalent cases avoided in year [per 100000] for Ireland

| **Prevalence cases avoided in year [per 100000] for Ireland** | | | | | |
| --- | --- | --- | --- | --- | --- |
| Year | Cancers | CHD & Stroke | Arthritis | Diabetes | Hypertension |
| **Scenario 1** |  |  |  |  |  |
| 2010 | -10 [+15] | 5 [+11] | -1 [+12] | 4 [+21] | -14 [+59] |
| 2020 | 31 [+17] | 20 [+14] | 4 [+12] | 93 [+21] | 344 [+62] |
| 2030 | 31 [+19] | 24 [+16] | 7 [+13] | 177 [+23] | 590 [+66] |
| **Scenario 2** |  |  |  |  |  |
| 2010 | -4 [+15] | 7 [+11] | 0 [+12] | -10 [+21] | -12 [+59] |
| 2020 | 93 [+17] | 80 [+14] | 30 [+12] | 298 [+21] | 881 [+62] |
| 2030 | 94 [+18] | 91 [+15] | 48 [+13] | 583 [+22] | 1450 [+65] |

**Incidence**

**Table S4:** Cumulative incidence cases from year 2010 [per 100000 of population in 2010] for Ireland

| **Cumulative incidence cases from year 2010 [per 100000 of population in 2010] for Ireland** | | | | | |
| --- | --- | --- | --- | --- | --- |
| Year | Cancers | CHD & Stroke | Arthritis | Diabetes | Hypertension |
| **Scenario 0** |  |  |  |  |  |
| 2010 | 252 [+4] | 525 [+6] | 42 [+2] | 137 [+3] | 913 [+9] |
| 2020 | 3038 [+15] | 5913 [+21] | 508 [+6] | 1650 [+11] | 10638 [+29] |
| 2030 | 6450 [+22] | 12134 [+31] | 1041 [+9] | 3568 [+17] | 21499 [+41] |
| **Scenario 1** |  |  |  |  |  |
| 2010 | 254 [+5] | 521 [+6] | 43 [+2] | 134 [+3] | 916 [+9] |
| 2020 | 2948 [+15] | 5801 [+21] | 499 [+6] | 1535 [+11] | 10199 [+28] |
| 2030 | 6291 [+22] | 11893 [+30] | 1021 [+9] | 3324 [+16] | 20675 [+40] |
| **Scenario 2** |  |  |  |  |  |
| 2010 | 249 [+4] | 524 [+6] | 44 [+2] | 139 [+3] | 916 [+9] |
| 2020 | 2803 [+15] | 5534 [+21] | 466 [+6] | 1268 [+10] | 9499 [+27] |
| 2030 | 5997 [+21] | 11329 [+29] | 957 [+9] | 2745 [+14] | 19360 [+38] |

**Table S5:** Cumulative incidence cases avoided from year 2010 [per 100000 of population in 2010] for Ireland

| **Cumulative incidence cases avoided from year 2010 [per 100000 of population in 2010] for Ireland** | | | | | |
| --- | --- | --- | --- | --- | --- |
| Year | Cancers | CHD & Stroke | Arthritis | Diabetes | Hypertension |
| **Scenario 1** |  |  |  |  |  |
| 2010 | -2 [+6] | 4 [+9] | -1 [+3] | 3 [+5] | -3 [+12] |
| 2020 | 90 [+22] | 112 [+31] | 9 [+9] | 115 [+16] | 439 [+41] |
| 2030 | 159 [+32] | 241 [+44] | 20 [+13] | 244 [+23] | 824 [+58] |
| **Scenario 2** |  |  |  |  |  |
| 2010 | 3 [+6] | 1 [+9] | -2 [+3] | -2 [+5] | -3 [+12] |
| 2020 | 235 [+22] | 379 [+30] | 42 [+9] | 382 [+15] | 1139 [+40] |
| 2030 | 453 [+32] | 805 [+43] | 84 [+13] | 823 [+22] | 2139 [+57] |
