# Supplementary material for: Application of the UK Foresight Obesity Model in Ireland: The Health and Economic Consequences of Projected Obesity Trends in Ireland
Source: PLoS One. 2013 Nov 13;8(11):e79827. doi: 10.1371/journal.pone.0079827 (PMC3827424; doi:10.1371/journal.pone.0079827)
Supplement: Appendix S5 — Healthcare costs by year, 2010-2030. Table S6, Scenario 0 – Obesity trends continue unabated (millions of euro). Table S7, Scenario 1- 1% decrease in population BMI. Table S8, Scenario 2- 5% decrease in population BMI. (DOCX) [file pone.0079827.s005.docx]

**Supplementary Information**

**Appendix 5:** Healthcare costs by year, 2010-2030 (Millions of euro)

**Table S6:** Scenario 0 – Obesity trends continue unabated (millions of euro)

| Year | Cancer | CHD & Stroke | Total |
| --- | --- | --- | --- |
| 2010 | 101 | 2265 | 2547 |
| 2011 | 118 | 2917 | 3220 |
| 2012 | 127 | 3242 | 3555 |
| 2013 | 131 | 3434 | 3753 |
| 2014 | 135 | 3557 | 3881 |
| 2015 | 138 | 3622 | 3952 |
| 2016 | 142 | 3710 | 4046 |
| 2017 | 143 | 3761 | 4100 |
| 2018 | 146 | 3847 | 4191 |
| 2019 | 151 | 3946 | 4297 |
| 2020 | 152 | 4014 | 4370 |
| 2021 | 155 | 4062 | 4425 |
| 2022 | 158 | 4196 | 4565 |
| 2023 | 161 | 4298 | 4673 |
| 2024 | 164 | 4324 | 4707 |
| 2025 | 167 | 4487 | 4878 |
| 2026 | 170 | 4577 | 4976 |
| 2027 | 172 | 4651 | 5056 |
| 2028 | 174 | 4739 | 5151 |
| 2029 | 177 | 4855 | 5273 |
| 2030 | 179 | 4969 | 5395 |

**Table S7:** Scenario 1- 1% decrease in population BMI

| Year | Cancer | CHD & Stroke | Total |
| --- | --- | --- | --- |
| 2010 | 101 | 2265 | 2547 |
| 2011 | 118 | 2856 | 3158 |
| 2012 | 125 | 3167 | 3478 |
| 2013 | 128 | 3347 | 3661 |
| 2014 | 134 | 3433 | 3753 |
| 2015 | 138 | 3479 | 3805 |
| 2016 | 141 | 3578 | 3908 |
| 2017 | 143 | 3675 | 4009 |
| 2018 | 146 | 3759 | 4097 |
| 2019 | 150 | 3845 | 4191 |
| 2020 | 153 | 3915 | 4266 |
| 2021 | 155 | 3977 | 4334 |
| 2022 | 157 | 4061 | 4423 |
| 2023 | 159 | 4173 | 4541 |
| 2024 | 163 | 4197 | 4572 |
| 2025 | 165 | 4329 | 4711 |
| 2026 | 168 | 4427 | 4816 |
| 2027 | 170 | 4509 | 4904 |
| 2028 | 172 | 4620 | 5021 |
| 2029 | 174 | 4773 | 5181 |
| 2030 | 176 | 4838 | 5252 |

**Table S8:** Scenario 2- 5% decrease in population BMI

| Year | Cancer | CHD & Stroke | Total |
| --- | --- | --- | --- |
| 2010 | 101 | 2265 | 2547 |
| 2011 | 112 | 2727 | 3022 |
| 2012 | 120 | 2997 | 3299 |
| 2013 | 125 | 3137 | 3443 |
| 2014 | 130 | 3199 | 3509 |
| 2015 | 133 | 3258 | 3571 |
| 2016 | 137 | 3348 | 3666 |
| 2017 | 141 | 3446 | 3769 |
| 2018 | 143 | 3472 | 3798 |
| 2019 | 145 | 3583 | 3912 |
| 2020 | 149 | 3641 | 3976 |
| 2021 | 151 | 3697 | 4036 |
| 2022 | 154 | 3790 | 4136 |
| 2023 | 156 | 3874 | 4224 |
| 2024 | 159 | 3957 | 4313 |
| 2025 | 160 | 4043 | 4403 |
| 2026 | 162 | 4152 | 4517 |
| 2027 | 164 | 4234 | 4606 |
| 2028 | 167 | 4331 | 4709 |
| 2029 | 170 | 4470 | 4854 |
| 2030 | 172 | 4510 | 4900 |
